# The Endoplasmic Reticulum Chaperone Protein GRP94 Is Required for Maintaining Hematopoietic Stem Cell Interactions with the Adult Bone Marrow Niche

**DOI:** 10.1371/journal.pone.0020364

**Published:** 2011-05-24

**Authors:** Biquan Luo, Ben S. Lam, Sung Hyung Lee, Shiuan Wey, Hui Zhou, Miao Wang, Si-Yi Chen, Gregor B. Adams, Amy S. Lee

**Affiliations:** 1 Department of Biochemistry and Molecular Biology and the USC Norris Comprehensive Cancer Center, University of Southern California Keck School of Medicine, Los Angeles, California, United States of America; 2 Department of Cell and Neurobiology, Eli and Edythe Broad Center for Regenerative Medicine and Stem Cell Research at USC, University of Southern California Keck School of Medicine, Los Angeles, California, United States of America; 3 Department of Molecular Microbiology and Immunology, University of Southern California Keck School of Medicine, Los Angeles, California, United States of America; Emory University School of Medicine, United States of America

## Abstract

Hematopoietic stem cell (HSC) homeostasis in the adult bone marrow (BM) is regulated by both intrinsic gene expression products and interactions with extrinsic factors in the HSC niche. GRP94, an endoplasmic reticulum chaperone, has been reported to be essential for the expression of specific integrins and to selectively regulate early T and B lymphopoiesis. In GRP94 deficient BM chimeras, multipotent hematopoietic progenitors persisted and even increased, however, the mechanism is not well understood. Here we employed a conditional knockout (KO) strategy to acutely eliminate GRP94 in the hematopoietic system. We observed an increase in HSCs and granulocyte-monocyte progenitors in the *Grp94* KO BM, correlating with an increased number of colony forming units. Cell cycle analysis revealed that a loss of quiescence and an increase in proliferation led to an increase in *Grp94* KO HSCs. This expansion of the HSC pool can be attributed to the impaired interaction of HSCs with the niche, evidenced by enhanced HSC mobilization and severely compromised homing and lodging ability of primitive hematopoietic cells. Transplanting wild-type (WT) hematopoietic cells into a GRP94 null microenvironment yielded a normal hematology profile and comparable numbers of HSCs as compared to WT control, suggesting that GRP94 in HSCs, but not niche cells, is required for maintaining HSC homeostasis. Investigating this, we further determined that there was a near complete loss of integrin α4 expression on the cell surface of *Grp94* KO HSCs, which showed impaired binding with fibronectin, an extracellular matrix molecule known to play a role in mediating HSC-niche interactions. Furthermore, the *Grp94* KO mice displayed altered myeloid and lymphoid differentiation. Collectively, our studies establish GRP94 as a novel cell intrinsic factor required to maintain the interaction of HSCs with their niche, and thus regulate their physiology.

## Introduction

In the adult hematopoietic system, hematopoietic stem cell (HSC) regulation of self-renewal and differentiation is at both the intrinsic and extrinsic level, and is tightly regulated under physiological conditions [1], [2]. HSCs reside in a specific anatomic location in the bone marrow (BM) known as the stem cell niche [3], [4]. Signaling cues from neighboring cells in the niche are key in dictating the function of the cell to maintain the hematopoietic system of the individual [5]–[7]. At the endosteal surface [8], which is the interface between bone and the BM, specialized osteoblasts represent the main components of HSC niche [9], [10]. It has been proposed that this heterogeneous group of cells regulates HSC survival, self-renewal, migration, differentiation, and quiescence [11], [12] through several soluble factors and their receptors such as angiopoietin/Tie2 [13], osteopontin [14], [15] and Ca^2+^/CaR [16], as well as direct contact through extracellular matrix and cell surface proteins [17], [18], such as integrins [19], [20]. HSC engraftment is a multistep process involving homing, transmarrow migration, and lodging within the BM niche [15], all of which is controlled by adhesion molecules, soluble ligands and their receptors [3].

It is also clear that the decision of the HSC to self-renew or differentiate is dependent upon the extrinsic signaling mechanisms controlling the expression of intrinsic determinants of HSC function. Previous studies have determined that a number of cell cycle regulators [1], [21] such as the early G1-phase checkpoint regulator p18^INK4C^ [22], and the later G1-phase checkpoint regulator P21^cip1/waf1^ [23], are critical to maintain HSC quiescence. Retinoblastoma family protein [24] and PTEN [25], [26] also play crucial roles in maintaining HSC homeostasis. Transcription factors such as Zinc-finger repressor Gfi1 [27], [28] and chromatin-associated factors like Bmi1 [29] have been implicated as key regulators in maintaining HSC self-renewal.

Glucose-regulated protein 94 (GRP94; also referred to as gp96, CaBP4, endoplasmin, Tra-1) is a metazoan-restricted member of the HSP90 protein family. It is traditionally regarded as an endoplasmic reticulum (ER) localized chaperone assisting in protein folding, assembly and secretion [30]. Due to its client selectivity and interactions with late folding intermediates, GRP94 is postulated to perform unique chaperone functions in the ER, and control specific pathways critical for cell growth and differentiation [31]. In Drosophila, Gp93 (ortholog of GRP94) is required for gut epithelial homeostasis and nutrient assimilation-coupled growth control, suggesting an essential role in the functional expression of specific secretory/integral membrane proteins in tissue specialization [32]. Loss of GRP94 function in mouse models revealed that it is required for mouse embryonic development [33] and plays an important role in immune functions [34]–[36]. For example, GRP94 is required for the expression of a large number of integrins, as well as Toll-like receptors and selectively regulates innate immunity of macrophages [37], [38], and early T and B lymphopoiesis [39], [40]. Furthermore, using a BM chimeric approach, an increase in HSCs and multipotent progenitors was observed in BM devoid of GRP94 [40], raising the interesting issue of how GRP94 deficiency could lead to HSC expansion.

Recently, we described the creation of a floxed mouse model of GRP94 targeting the exon 2 of the *Grp94* allele (*Grp94^F/F^*) for examining GRP94 function in specific tissues [41], [42]. We report here that by breeding the *Grp94^F/F^* mice with the transgenic mice expressing *Mx-1* promoted Cre recombinase [43], we generated *Grp94^F/F^*; *Mx-1-Cre* (referred to below as cKO) mice that allows inducible, acute homozygous knockout (KO) of GRP94 in the hematopoietic system through injection of polyinosinic-polycytidylic acid (pI.pC). GRP94 deficiency results in a striking 2-fold expansion of the HSC-enriched Lin^−^ c-Kit^+^ Sca-1^+^ (LSK) cell population (p<0.001) with increases in both the long-term (LT) and short-term (ST) HSC populations. Cell cycle analysis showed loss of quiescence and lodgment assays revealed impaired HSC-niche interaction as a major contributing factor for this increase. Through transplantation assays we further determined that this is a HSC cell autonomous effect, which can be mediated by the loss of integrin α4 expression on the cell surface of *Grp94* KO HSCs, leading to impaired HSC retention in the niche. Collectively, our studies establish GRP94 as a novel cell intrinsic factor required to maintain the interaction of HSCs with their niche, and thus regulate their physiology.

## Results

### Creation of a mouse model with inducible knockout of *Grp94* in the hematopoietic system

**Figure 1:**
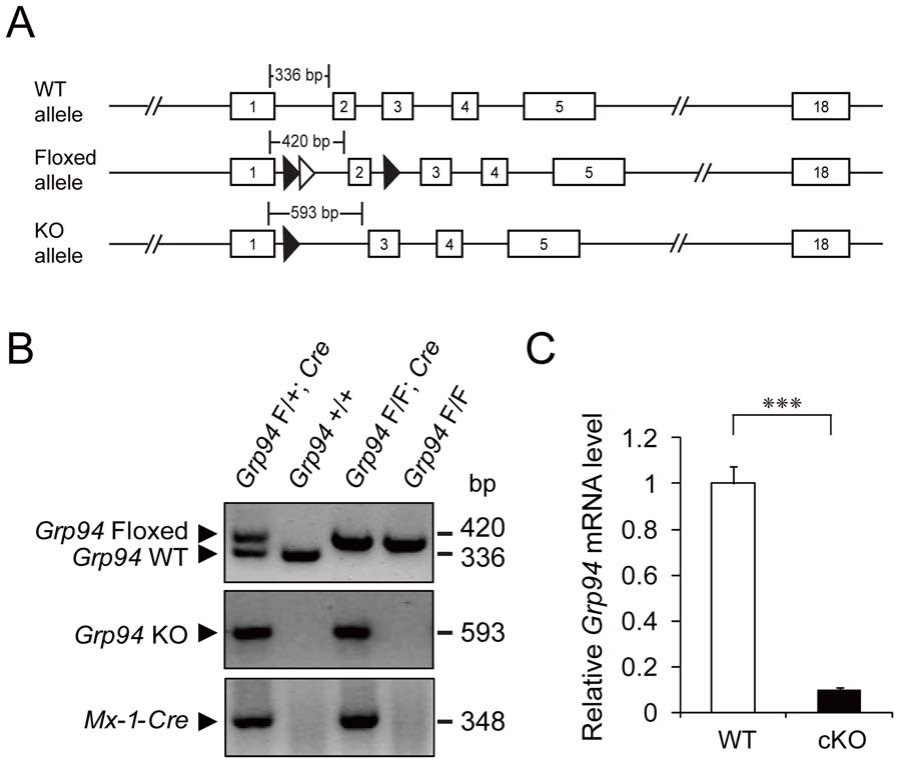
Conditional knockout of *Grp94* in the bone marrow. A) Schematic drawings of the *Grp94* wild-type (WT) allele, the floxed allele and the knockout (KO) allele. The exons are boxed and numbered. The loxP sites (closed triangle) and the FRT site (open triangle) and expected PCR products for genotyping is indicated. B) Representative BM PCR genotyping results of mice with indicated genotypes after pI.pC injection. C) *Grp94* mRNA expression measured by quantitative real-time PCR from WT (n = 16) and cKO (n = 18) mouse BM after pI.pC injection. The level of *Grp94* mRNA was normalized against the level of internal control *18S RNA*. The data are presented as mean ± s.e., ***p<0.001.

To study the *in vivo* function of GRP94, we created *Grp94* mutant mice with the floxed or knockout allele (Figure 1A). Deletion of exon 2 flanked by the loxP-FRT sites led to an early frameshift mutation resulting in the inactivation of the *Grp94* allele. To determine the role of GRP94 in the hematopoietic system, we bred *Grp94^F/F^* mice with transgenic mice bearing the *Mx-1-Cre* transgene that allows GRP94 to be acutely eliminated in the hematopoietic system in an inducible manner by administration of pI.pC. In these studies, *Grp94* was deleted in 5.5 to 6.5 week old *Grp94^F/F^*; *Mx-1-Cre* (referred to below as cKO) mice. Littermates lacking the *Cre* transgene (*Grp94^F/F^*), which are phenotypically equivalent to animals with wild-type (WT) *Grp94* alleles, were also injected with pI.pC and served as controls for any side effects of pI.pC injection. These control mouse cohorts are referred to below as WT mice. The status of the *Grp94* allele deletion was validated by PCR in isolated BM cells (Figure 1B). Real-time quantitative PCR analysis with mouse BM cells 11 days after 7 shots of pI.pC injection showed that *Grp94* transcripts were mostly depleted from the BM cells of the cKO mice (Figure 1C).

### GRP94 deficiency expands primitive cell pool through loss of quiescence

**Figure 2:**
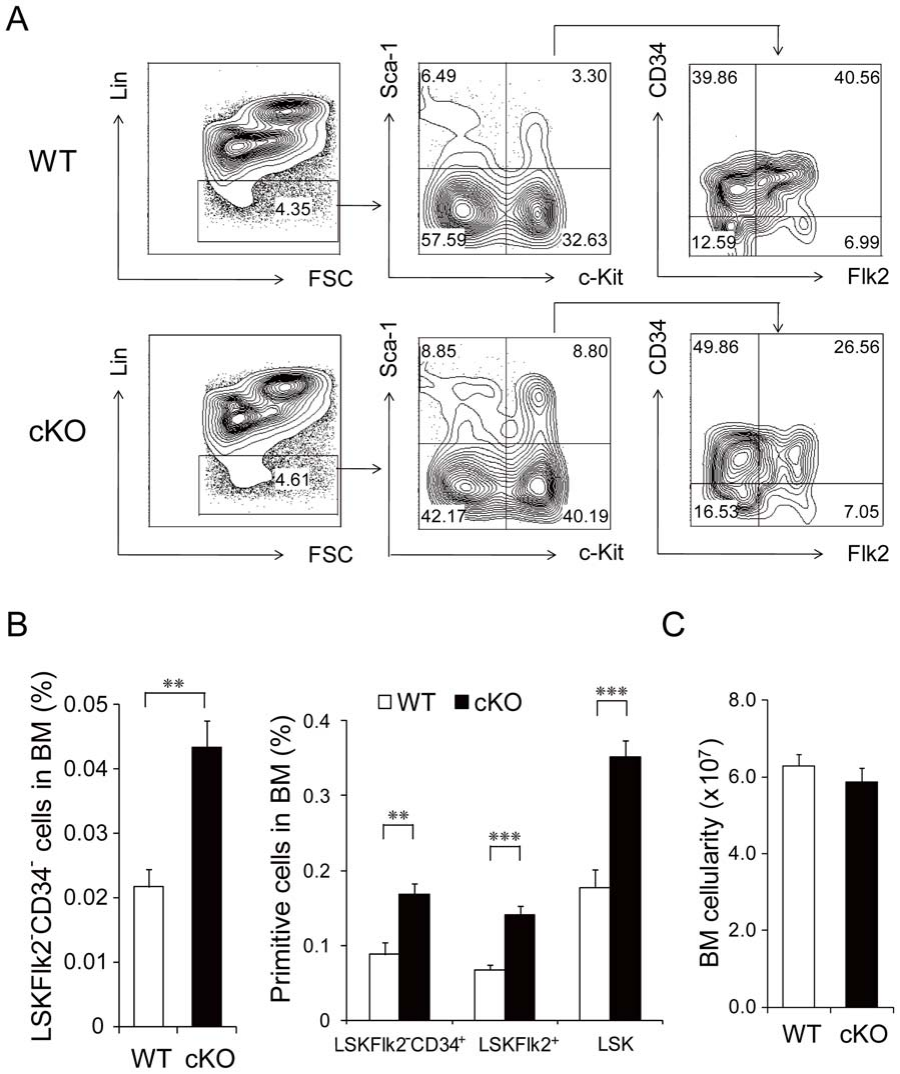
GRP94 deficiency in the bone marrow expanded the primitive cell pool. A) Representative flow cytometric analysis with BM cells using Lin, c-Kit, Sca-1, Flk2 and CD34. B) Quantitation of flow cytometric analysis of primitive cell proportions. Left panel shows the percentage of LSKFlk2^−^CD34^−^ in BM and right panel shows LSKFlk2^−^CD34^+^, LSKFlk2^+^ (n = 5 for WT, n = 8 for cKO) and total LSK (n = 16 for WT, n = 22 for cKO) cells in BM using Lin, c-Kit, Sca-1. C) Total BM cell number from WT (n = 19) and cKO (n = 20) mice (p = 0.373). All data are presented as mean ± s.e., **p<0.01, ***p<0.001.

**Figure 3:**
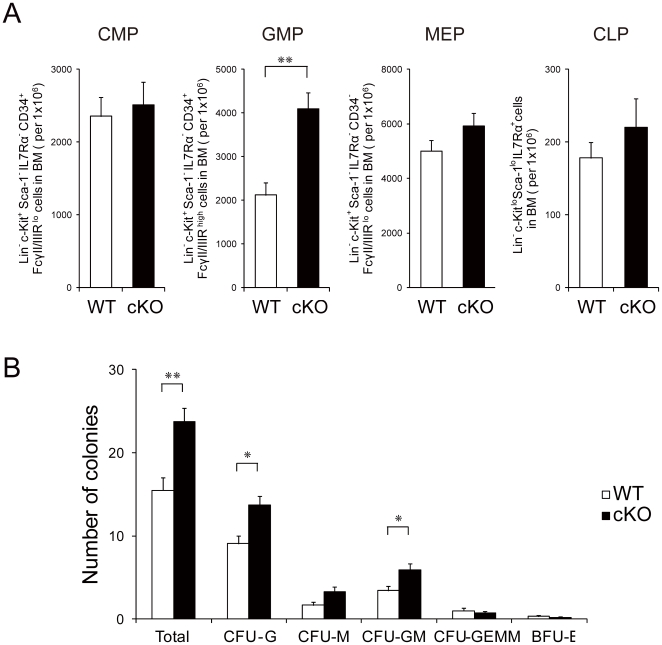
GRP94 deficiency led to increased granulocyte–monocyte progenitors in the bone marrow. A) Quantitation of flow cytometric analysis of myeloid and lymphoid progenitors including common myeloid progenitor (CMP), granulocyte-monocyte progenitor (GMP), megakaryocyte-erythroid progenitor (MEP) and common lymphoid progenitor (CLP) from WT (n≥7) and cKO (n≥10) mice. B) Quantitation of colonies formed from unfractioned BM from WT (n = 4) and cKO (n = 7) mice in methylcellulose medium. All data are presented as mean ± s.e., *p<0.05, **p<0.01.

In analyzing the effect of acute *Grp94* deletion on primitive hematopoietic cells, we observed that within 3 weeks of GRP94 depletion there was an expansion of the most primitive Lin^−^ c-Kit^+^ Sca-1^+^ Flk2^−^ CD34^−^ (LSKFlk2^−^CD34^−^) LT-HSC-enriched cell population [44] (2-fold, p<0.01) in the BM. Correlating with this was an expansion of the ST-HSC enriched LSKFlk2^−^CD34^+^ cells, MPP-enriched LSKFlk2^+^ and LSK populations of cells (p<0.001) (Figure 2A and 2B), indicating that all primitive hematopoietic cells were significantly increased upon acute hematopoietic *Grp94* deletion. These findings, coupled with the observation that the total BM cell number was not significantly different in the mutant mice (p = 0.373) (Figure 2C), reveal that the total number of primitive hematopoietic cells in cKO mice BM is higher compared to controls. We also analyzed the progenitor cell populations in the BM. While the common myeloid progenitor (CMP) enriched Lin^−^ c-Kit^+^ Sca-1^−^ IL-7Rα^−^ CD34^+^ FcγII/IIIR^lo^ cells, the common lymphoid progenitor (CLP) enriched Lin^−^ c-Kit^lo^ Sca-1^lo^ IL-7Rα^+^ cells, and the megakaryocyte-erythroid progenitor (MEP) enriched Lin^−^ c-Kit^+^ Sca-1^−^ IL-7Rα^−^ CD34^−^ FcγII/IIIR^lo^ cells were all comparable between the cKO mice and their WT littermates, there were significantly more granulocyte-monocyte progenitor (GMP) enriched Lin^−^ c-Kit^+^ Sca-1^−^ IL-7Rα^−^ CD34^+^ FcγII/IIIR^high^ cells in the cKO mice (Figure 3A). This correlates with the increased number of colonies (p<0.01 for total colony number; p<0.05 for CFU-G and CFU-GM; p = 0.07 for CFU-M) formed by unfractioned cKO BM cells in CFC assays (Figure 3B).

**Figure 4:**
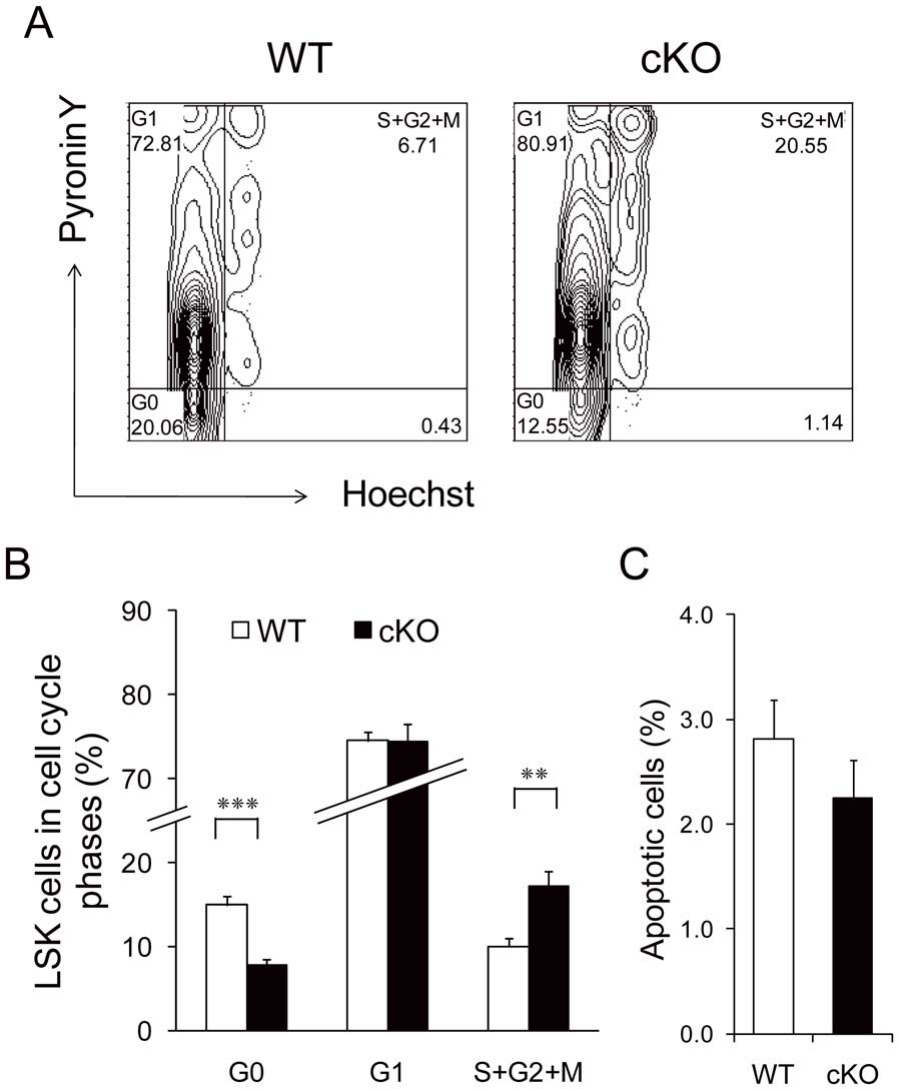
GRP94-deficient LSK cells displayed increased proliferation and loss of quiescence. A) Representative flow cytometric analysis of LSK cell cycle status by Hoechst and Pyronin Y staining. To examine early effects of GRP94 depletion on HSC proliferation, BM was extracted from WT and cKO mice 3 days after 4 shots of pI.pC injection every other day. B) Summary of cell cycle distribution of LSK cells from WT and cKO mice (n = 7). C) Summary of flow cytometric analysis of apoptotic LSK cells using Annexin V and 7AAD (n = 5 for WT, n = 8 for cKO) (p = 0.324). All data are presented as mean ± s.e., **p<0.01, ***p<0.001.

To examine the underlying cause for the increase in primitive hematopoietic cells in the BM of the cKO mice, we analyzed the cell cycle distribution in LSK cells by Hoechst/Pyronin Y staining (Figure 4A). While approximately 75% of the LSK cells in both genotypes were in G1 phase, the proportion of cells in G0 decreased from 15% in the control to 7.7% in the cKO mice (p<0.001), whereas the amount of S+G2+M cells increased from 10% in the control to 17% for cKO mice (p<0.01) (Figure 4B). Furthermore, the expansion of the *Grp94* KO primitive hematopoietic cell pool was not a consequence of altered cell survival as analysis of the level of apoptotic cells using Annexin V did not reveal significant differences between WT and cKO LSK cells (p = 0.324) (Figure 4C). These findings suggest a loss of quiescence and increase in proliferation account at least in part for the primitive hematopoietic cell expansion observed in cKO mice, similar to what has been shown using other models of HSC function [45].

### *Grp94* KO HSCs display increased mobilization and impaired HSC-niche interaction

**Figure 5:**
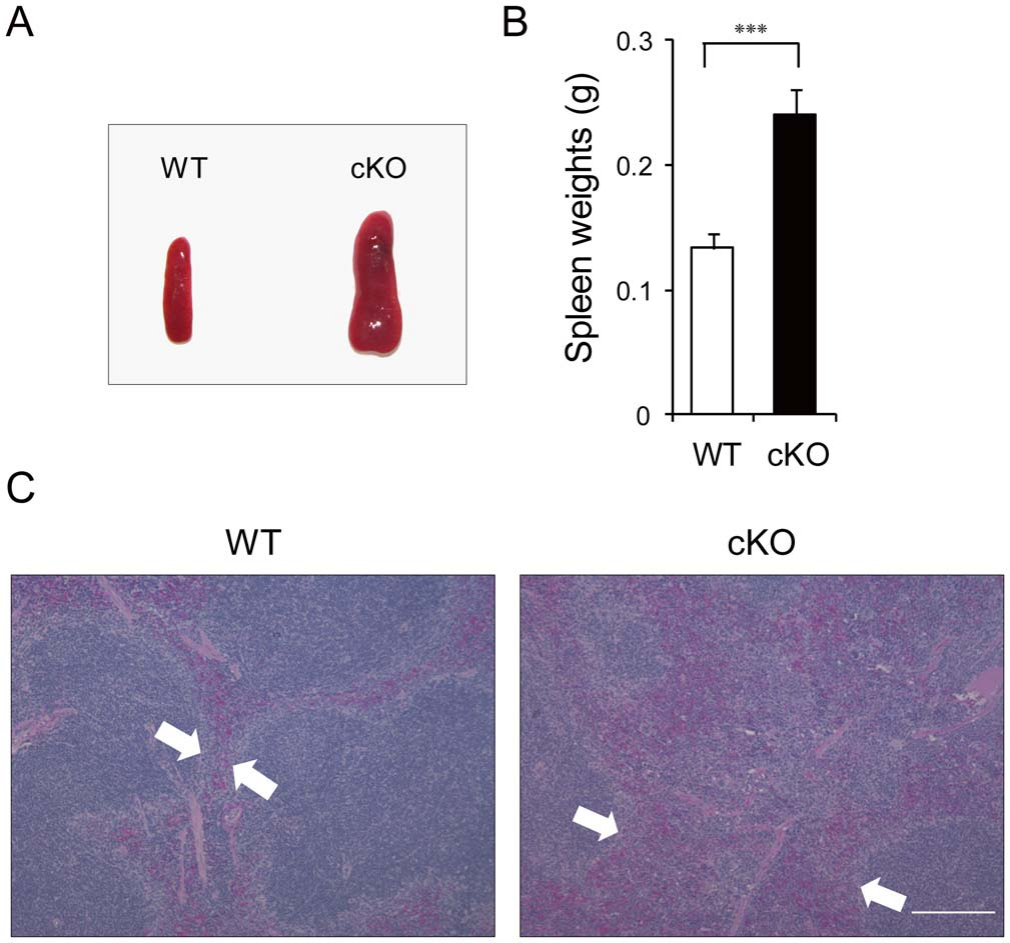
Increase extramedullary hematopoiesis in the spleen of *Grp94* KO mice. A) Representative spleen size and morphology of WT and cKO mice. B) Average spleen weights of WT (n = 19) and cKO (n = 25) mice. The data are presented as mean ± s.e., ***p<0.001. C) Representative H&E staining of paraffin sections of spleen from WT and cKO mice. Hematopoietic cells in the red pulp are indicated by the area between the two arrows. Scale bar represents 2 mm.

**Figure 6:**
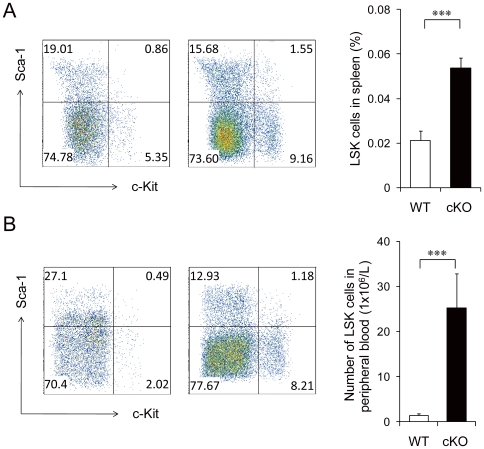
Increased mobilization of *Grp94* KO HSCs. A) Representative flow cytometric analysis of splenocytes from WT (n = 9) and cKO (n = 9) mice using Lin, c-Kit and Sca-1 (left) and quantitation (right). In these studies, splenocytes were extracted from WT and cKO mice 5 days after 7 injections of pI.pC (to examine HSC mobilization before the spleen was enlarged). B) Representative flow cytometric analysis of blood MNCs from WT (n = 11) and cKO (n = 10) mice using Lin, c-Kit and Sca-1 (left) and quantitation (right). All data are presented as mean ± s.e., ***p<0.001.

We next examined whether the alteration in the cell cycle status of the primitive hematopoietic cells was accompanied by any changes in their localization [46]. Histological analysis showed that the spleen size was significantly enlarged in cKO mice (Figure 5A and 5B) with signs of increased extramedullary hematopoiesis as indicated by the increased hematopoietic cells in the red pulps (Figure 5C). Flow cytometric analysis showed that there was a significant increase (2.5-fold, p<0.001) in the percentage of LSK cells in the spleen of cKO mice compared to WT mice (Figure 6A). The number of circulating LSK cells in the peripheral blood of cKO mice were also greatly increased (17-fold, p<0.001) compared to control (Figure 6B). We also observed increased Lin^−^ Sca-1^−^ c-Kit^+^ progenitor cells in the peripheral circulation. These results suggest that primitive cell mobilization was increased as a consequence of *Grp94* deletion.

**Figure 7:**
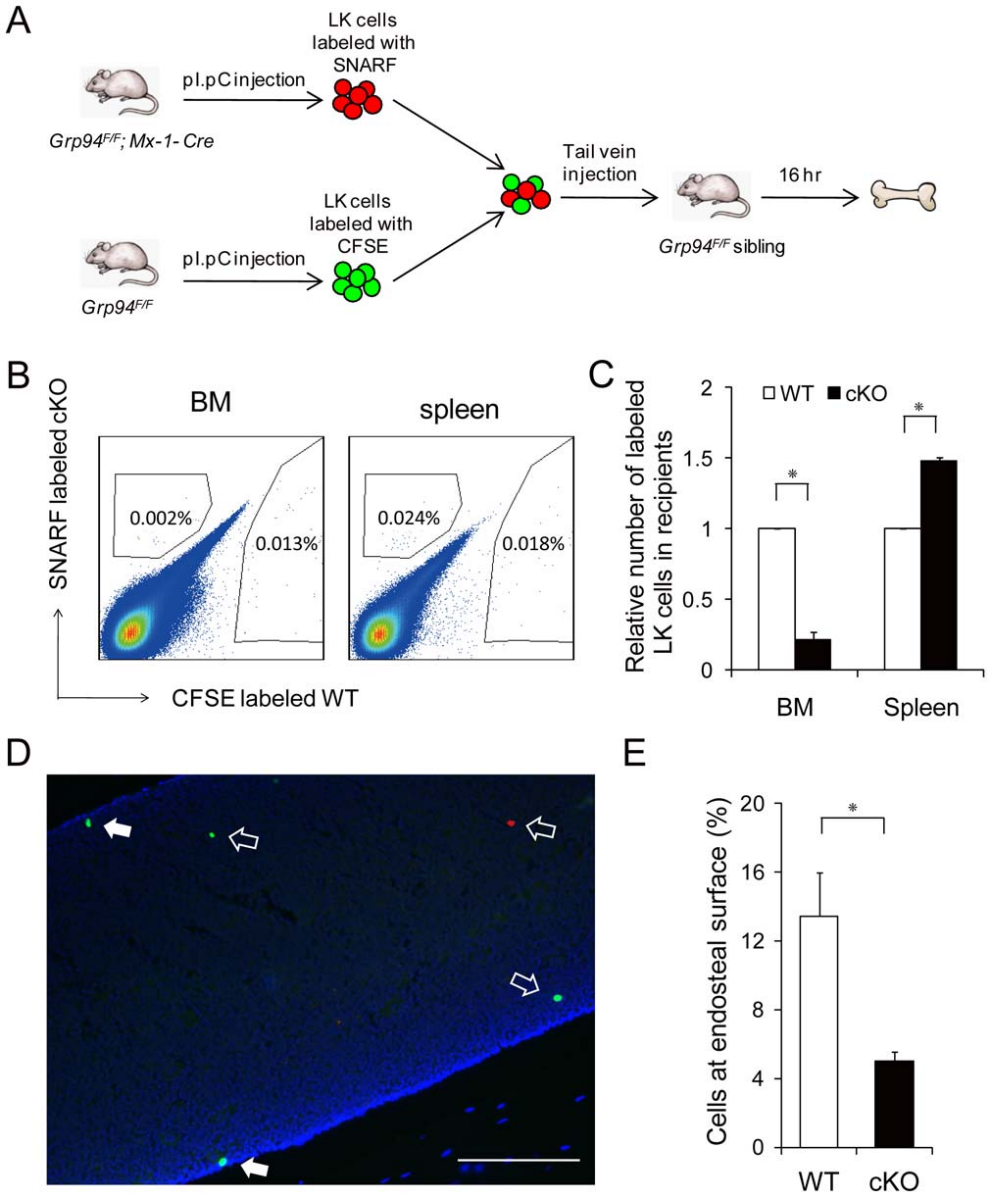
*Grp94* KO HSCs displayed impaired interaction with the niche. A) Scheme of the *in vivo* competitive lodgment assay. B) Representative flow cytometric analysis of CFSE-labeled WT LK cells and SNARF-labeled cKO LK cells with host BM and spleen cells. C) Summary of LK cells homed to the BM and spleen (n = 4 for BM, n = 2 for spleen), with the level of WT cells in BM and spleen set as 1. D) Bone section of a recipient femur. WT LK cells were labeled with CFSE (green); cKO LK cells were labeled with SNARF (red); and nuclei were stained with DAPI (blue). Solid arrows indicate cells lodged in the endosteal region (within two cell diameters from the endosteal surface), while open arrows indicate cells located in the central marrow. Scale bar represents 1 mm. E) Summary of the percentage of labeled LK cells found at the endosteal region among those homed to BM from 3 independent experiments. All data are presented as mean ± s.e., *p<0.05.

**Figure 8:**
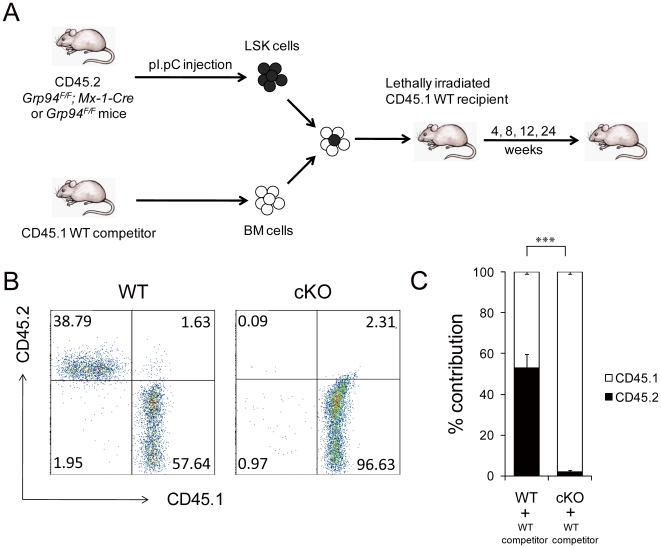
*Grp94* KO HSCs failed to reconstitute the hematopoietic system in the presence of WT competitors. A) Scheme of the *in vivo* competitive repopulation assay. B) Representative flow cytometric analysis with tail blood from recipient mice 4 weeks after BM transplantation. CD45.1^+^ cells represent blood cells from WT competitor, while CD45.2^+^ cells represent progenies from WT or cKO LSK cells. Tail blood from recipient mice 8, 12, and 24 weeks after BM transplantation yielded the same results (data not shown). C) Summary of the percentage contribution of WT or cKO LSK cells to the peripheral blood MNCs (n = 5). The data are presented as mean ± s.e., ***p<0.001.

Since the HSC niche has been proposed to maintain stem cells in the BM in a quiescent state, we hypothesized that the increase in proliferation of the *Grp94* KO HSCs may have been mediated by a loss of interaction with the niche, which thus also led to increased HSC mobilization and enlargement of the spleen by extramedullary hematopoiesis in cKO mice. Therefore, we directly examined the capability of *Grp94* KO progenitors to home and lodge in the endosteal niche. To do this, we performed a competitive lodgment assay (Figure 7A), in which the lodging of primitive cells is dependent upon their functional interaction with the niche *in vivo*. An equal number of WT and cKO Lin^−^ c-Kit^+^ (LK) cells were labeled with fluorescent vital dyes CFSE and SNARF respectively, and co-injected into non-irradiated WT littermates. In these experiments we used LK cells, as in agreement with previous studies, the use of a purified HSC population would result in too few cells to be reliably detected [47]. We observed that fewer labeled cells from mutant mice homed to BM, while more of them migrated to the spleen of recipient mice (p<0.05) (Figure 7B and 7C). We cut a total of 180 sections of femurs and tibias from a total of three recipient mice in three independent experiments, and visualized the localization of cells as shown in Figure 7D. The endosteal region is defined as the area within 2 cell diameters from endosteal surface. Among all the 400–1000 cells that homed to the BM (numbers varied each time according to the number of cells injected), about 13.5% of WT LK cells lodged at the endosteal region, while only 5% of the cKO cells that homed to the BM were found at the endosteal surface (p<0.05) (Figure 7E). These findings suggest that acute deletion of *Grp94* in primitive hematopoietic cells impairs the ability of HSCs to home to BM and lodge at the endosteal niche. As a consequence, in the competitive repopulation assay (Figure 8A) cKO LSK cells failed to engraft and reconstitute the hematopoietic system in lethally irradiated recipient mice in the presence of WT competitors (Figure 8B and 8C).

### GRP94 in HSCs, but not niche cells, is necessary for maintaining HSC homeostasis

**Figure 9:**
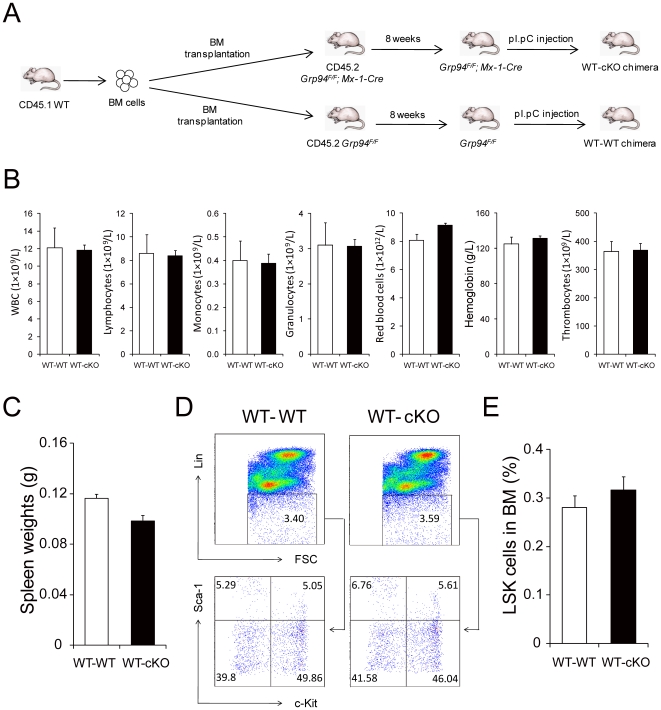
Effect of cKO microenvironment on HSC maintenance. A) Scheme of creating chimeric mice with WT hematopoietic cells transplanted into WT or cKO microenvironment. B) Complete blood count with tail peripheral blood from WT-WT (n = 5) and WT-cKO (n = 8) chimeric mice. C) Quantitation of spleen weights from WT-WT (n = 4) and WT-cKO (n = 5) chimeric mice. D) Representative flow cytometric analysis with BM from WT-WT and WT-cKO chimeric mice using Lin, c-Kit and Sca-1. E) Summary of percentage of LSK cells in the BM from WT-WT (n = 5) and WT-cKO (n = 8) chimeric mice. All data are presented as mean ± s.e..

In the competitive repopulation and competitive lodgment assay, where cKO cells are transplanted into a WT microenvironment, we observed impairment in the function of the cells indicating that *Grp94* deletion in HSCs at least partially contributes to the disruption of the HSC-niche interaction. However, in our model system *Grp9*4 was knocked out in the whole BM, therefore the disruption of HSC-niche interactions could also be attributed to the niche cells. To further explore a possible extrinsic role of GRP94 null niche cells in HSC homeostasis disturbance in cKO mice, we created a chimeric mouse model with WT hematopoietic cells and a GRP94 null microenvironment. WT BM cells were transplanted into lethally irradiated WT or cKO recipient mice. Following 8 weeks to allow for full reconstitution of WT hematopoietic system, *Grp94* deletion was induced by pI.pC administration (Figure 9A). According to previous reports, BM stromal cells do not engraft after BM transplantation [48], therefore, *Grp94* KO only occurred in the hematopoietic cells. Chimeras with WT hematopoietic cells and cKO microenvironment showed a normal hematology profile and spleen size as WT control chimeras (Figure 9B and 9C). Flow cytometric analysis revealed a similar LSK percentage in BM from cKO chimeras and WT controls (Figure 9D and 9E). These findings provide direct evidence that GRP94 in HSCs is required for their interaction with the niche, and depleting GRP94 in BM microenvironment itself is not sufficient to affect the regulation of HSCs.

### *Grp94* KO HSCs lack cell surface integrin α4 expression and exhibit impaired binding to fibronectin

**Figure 10:**
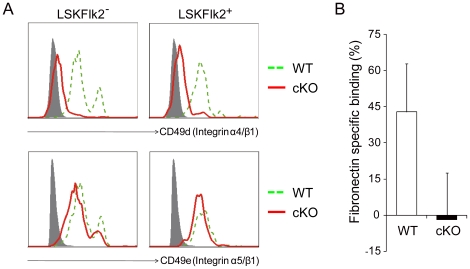
Inability of *Grp94* KO HSCs to express surface integrin α4 and bind to fibronectin. A) Representative flow cytometric analysis of CD49d and CD49e with BM LSKFlk2^−^ and LSKFlk2^+^ cells from WT and cKO mice. Grey-filled histogram represents isotype control staining; dashed green line represents WT cells; solid red line indicates cKO cells. B) The percentage of WT and cKO LSK cells bound to fibronectin *in vitro*. The number of cells binding to BSA was subtracted from that binding to fibronectin, the results then were normalized against the number of WT cells bound to BSA. The experiments were performed twice in duplicate; each replicate contained pooled BM from 2 to 4 WT or cKO mice. The data are presented as mean ± s.e..

To investigate the potential mechanism by which GRP94 maintains the interaction between HSCs and the niche, we examined the cell surface expression of integrin α4 and α5, which are known to be important for the homing and adhesion of HSCs to the endosteal niche [49], [50]. Flow cytometric analysis with mouse BM LSKFlk2^−^ and LSKFlk2^+^ populations demonstrated significantly lower expression of cell surface CD49d (integrin α4/β1) on cKO than WT cells, whereas CD49e (integrin α5/β1) expression was comparable between the two groups (Figure 10A). These results establish that GRP94 is required for the expression of integrin α4 on the cell surface of hematopoietic stem and progenitor cells, which is consistent with the recent finding that loss of GRP94 abrogates integrin α4 but not α5 expression on BM cells [40].

To test specific interactions of the primitive hematopoietic cells with adhesion molecules known to be present at the endosteal surface and play a role in mediating HSC-niche interactions, we examined the ability of the cells to bind to fibronectin *in vitro*, the binding partner of integrin α4, with binding to BSA as a negative control. While WT LSK cells were able to bind to fibronectin, we did not detect binding of the cKO cells to fibronectin above the negative control level (Figure 10B). Collectively, these findings demonstrate that acute depletion of GRP94 in HSCs disrupts the interaction of HSCs with niche, possibly through the disruption of adhesion to key extracellular matrix molecules mediated by integrin α4.

### Deletion of *Grp94* in the hematopoietic system leads to altered myeloid and lymphoid differentiation

**Figure 11:**
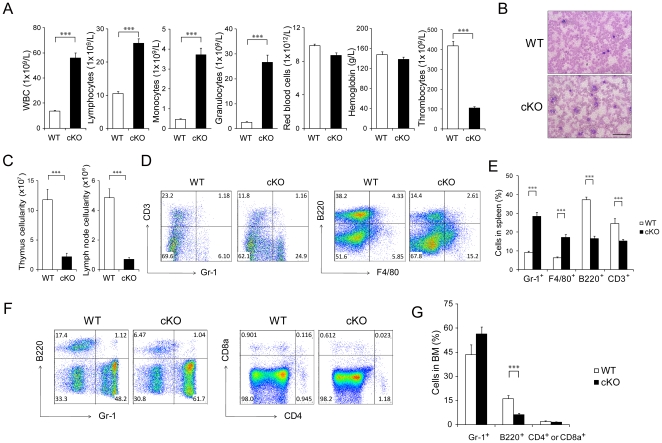
*Grp94* KO mice displayed altered myeloid and lymphoid differentiation. A). Complete blood count of peripheral blood from WT (n = 31) and cKO (n = 37) mice. B) Representative Wright-Giemsa staining of blood smear with tail peripheral blood from WT and cKO mice. Scale bar represents 500 µm. C) Total thymus cell number (left) and total left and right axillary lymph nodes cell number (right) from WT and cKO mice (n = 7 for each group). D) Representative flow cytometric analysis of splenocytes from WT and cKO mice using lineage markers Gr-1 and CD3 (left), F4/80 and B220 (right). E) Quantitation of (D) from WT (n = 4) and cKO (n = 7) mice. F) Representative flow cytometric analysis with BM cells using lineage markers Gr-1 and B220 (left) and CD4 and CD8a (right). G) Quantitation of (F). Gr-1 and B220 (n = 7 for WT and n = 9 for cKO mice); CD4 and CD8a (n = 7 for each genotype). All data are presented as mean ± s.e., ***p<0.001.

To determine the consequence of acute depletion of GRP94 following Mx-1-Cre induction, we examined the hematopoietic phenotypes of cKO mice compared to control siblings. Complete blood count analysis demonstrated a general leukocytosis and severe thrombocytopenia, while red blood cell and hemoglobin counts were normal. Monocytes and granulocytes in peripheral blood were increased 8-fold and 10-fold, respectively, in cKO mice compared to control mice (Figure 11A), which is consistent with the increased GMP in the cKO BM (Figure 3A). Analysis of blood smears further confirmed the leukocytosis in peripheral blood (Figure 11B). Compared with WT littermates, cKO mice showed significantly lower thymus and lymph node cellularities (Figure 11C), consistent with smaller thymus and peripheral lymph nodes (data not shown). The increase in myeloid cells and decrease in lymphoid cells corresponded with a higher proportion of granulocytes and macrophages and a lower proportion of T-cells and B-cells in the spleen of the cKO mice (Figure 11D and E). The expansion of myeloid cells was also evident in the BM, where Gr-1^+^ cells were increased and B220^+^ cells were decreased in cKO mice. The proportion of CD4^+^ and CD8^+^ cells in the BM of cKO mice were comparable to the control mice (Figure 11F and G).

## Discussion

GRP94 is the most abundant glycoprotein in the ER and is emerging as an important player in protein processing. Its unique interaction with specific client proteins may allow it to specifically regulate cellular processes distinct from other major chaperones in the ER. In particular, recent model studies suggested that GRP94 is critically required for the functional expression of secretory and/or membrane proteins that enable the integration of cells into tissues [32]. Here we examined the role of GRP94 in the HSC interactions with the adult BM niche. The model system that we employed is the *Grp94*; *Mx-1-Cre* mouse model whereby GRP94 can be acutely eliminated largely in the hematopoietic system in a temporally controlled manner in adult mice. Our results showed that within 3 weeks of depletion of GRP94 in the hematopoietic cells, there was a 2-fold increase in primitive HSCs in the mutant mice. The *Grp94* KO LSK cells are more proliferative, likely as a consequence of loss of niche interaction. The latter is supported by our observation that the mutant LSK cells are more mobilized to the spleen and peripheral blood. The mutant LSK cells lack cell surface integrin α4 expression and do not bind to fibronectin as efficiently as the WT LSK cells *in vitro*, are less able to lodge in the BM niche *in vivo*, and as a consequence showed no engraftment in competitive repopulation assays.

The maintenance of HSCs involves the balance between self-renewal and differentiation, proliferation and quiescence, mobilization and homing, as well as their capacity to provide lifelong reconstitution of all blood-cell lineages after transplantation into lethally irradiated recipients [3], [12]. Our study demonstrates the unique function of GRP94 in regulating the homeostasis of HSCs and their interaction with the stem cell niche. Currently, the regulation of HSC physiology is mostly attributed to transcription factors, cell-cycle regulators, chromatin-associated proteins, extracellular signaling molecules and their receptors [1]. Here we explore the notion that an ER chaperone protein may play a role in the regulation of HSC-niche interactions. The HSC niche is believed to house HSCs, maintain their quiescence and prevent them from differentiation [3]. Here we determined that *Grp94* KO primitive cells are less efficient in contacting with BM niche, with the consequence that the mutant LSK cells can exit quiescence and enter cell cycle. While the precise mechanisms by which GRP94 affects this awaits future investigation, one plausible mechanism may be attributed to the requirement of GRP94 to facilitate processing of factors such as integrins specifically required for HSC-niche adhesion [19]. Secretory factors and cell-surface proteins are synthesized in the rough ER, and translocated into the ER where they are folded into their functional conformation by ER chaperone proteins [30]. GRP94 has been reported to be essential for the proper folding of several integrins, including α4 [40]. This has been reported to mediate the interaction of HSCs with niche cells and the extracellular matrix, as loss of integrin α4 results in impaired homing and reduced efficiency of HSCs to lodge to the endosteal surface [19], [49]. While integrin α4 and α5 are both important for HSC retention in the niche [17], [51], GRP94 is specifically required for processing integrin α4 but not α5 in HSCs. The failure of the *Grp94* KO HSCs to express integrin α4 on the cell surface and its inability to bind to fibronectin are supportive of this notion. It is also possible that GRP94 is required for the proper folding of soluble ligands mediating the cross talk from HSCs to the niche. Other mechanisms may include cell cycle regulation by GRP94 and the ability of GRP94 to regulate ER Ca^2+^ efflux and signaling. As GRP94 is also recently localized to the cell surface and also identified as a transmembrane ER protein [30], it may also control signaling pathways initiated from the cell surface and/or the ER and cytosol interphase affecting stem cell proliferation.

Our studies raise the general issue concerning the roles of protein folding chaperones in hematopoiesis and its relationship to the unfolded protein response (UPR). Hematopoiesis is tightly regulated by growth factors, cytokines and their receptors, which are secretory and cell membrane proteins [52]. Secretory factors and cell surface proteins are synthesized in the rough ER where they are folded into their functional conformation by ER chaperone proteins. Therefore, ER chaperone proteins are likely to be important for maintaining hematopoiesis, and their function depends on the role of specific client proteins in hematopoiesis. Other than synthesizing client proteins that are regulating hematopoiesis, the ER is also involved in hematopoiesis through UPR signaling pathways. For example, UPR sensor IRE1 ribonuclease activity for XBP-1 splicing is required for both the early differentiation of T and B lymphocytes and the terminal differentiation of activated B cells to immunoglobin-secreted plasma cells [53], [54]. Whether UPR signaling is involved in regulating HSC homeostasis is not known, and awaits further investigation.

In comparing our Mx-1-Cre-mediated acute GRP94 KO model with the chimera model using tamoxifen-inducible GRP94 KO following BM transplantation [40], similar hematopoietic phenotypes, including general leukocytosis, increased spleen cellularity, decreased thymus and lymph node cellularities, increased myeloid cells and decreased lymphoid cells in the BM and spleen were observed in both models. The increase of LSK cells in the BM, spleen and peripheral blood and loss of integrin α4 expression upon GRP94 depletion are also in agreement with the previous report [40], which focuses on the role of GRP94 in lymphogenesis, specifically pro-B to pre-B transition in B cell development, as well as T cell development beyond CD4^−^ CD8^−^ double negative stage. Our work here investigates the unique function of GRP94 in the homeostasis of HSCs and their interaction with the stem cell niche. With our model, we provide new evidence that the expansion of primitive hematopoietic cells is attributed to the change of cell cycle status upon *Grp94* deletion and that the change of HSC cell cycle status and localization is due to loss of interaction with the niche, revealed by the impaired homing and lodging ability of GRP94-deficient HSCs *in vivo*. We further determined that GRP94 in the niche cells is not required for maintaining the interaction with HSCs.

In summary, we identified GRP94 as a novel regulator of HSCs physiology and their interaction with the niche. It is tempting to speculate that agents that can alter GRP94 expression or activity may be explored for therapeutic intervention, such as increasing the proportion of HSCs in peripheral blood as a source for blood stem cell therapy, or as an alternative method for non-myeloablative conditioning through emptying the HSC niche [55] and these warrant future investigation. Furthermore, overexpression of GRP94 has been associated with cellular transformation, tumorigenicity and decreased sensitivity to anti-cancer treatment [56]. With cancer stem cells implicated as the cancer initiating cells responsible for tumorigenesis and contribute to cancer resistance [57], it would be interesting to determine in future studies whether GRP94 regulates cancer stem cell niche interaction and thus contributes to tumorigenicity and self renewal of resistant cancer cells.

## Materials and Methods

### Ethics statement

All protocols for animal use were reviewed and approved by the USC Institutional Animal Care and Use Committee. The animal assurance number is A3518-01. The protocol number is 9964.

### Mice

*Grp94^F/F^* mice in a mixed C57BL/6; 129/Sv background were generated as previously described [42]. *Grp94^F/F^* mice were crossed with the transgenic *Mx-1-Cre* mice on a C57BL/6 background (Jackson Laboratory) to generate *Grp94^F/F^*; *Mx-1-Cre* mice. Littermates that were negative for *Cre* transgene were used as controls. Genotyping was performed by PCR using genomic DNA extracted from mouse tail biopsies as previously described [58]. To induce the genomic deletion, 5.5 to 6.5 week-old male and female mice were injected intraperitoneally with pI.pC (25 µg/g mouse body weight) every other day. Mice were analyzed 11 days post 7 injections, except with modifications as specifically mentioned in the figure legends.

### Complete blood count

Peripheral blood was collected via tail bleeding and analyzed using an auto hematology analyzer BC-2800 vet (Mindray, Redmond, WA) according to manufacturer's instructions.

### Flow cytometry

BM cells were flushed from long bones (tibias and femurs) with Dulbecco's phosphate-buffered saline without calcium and magnesium (DPBS), and then filtered through nylon screen (70 micron, BD Biosciences) to obtain single cell suspension. Splenocytes were collected following disaggregation of the spleens in DPBS, with red blood cells (RBCs) lysed in lysing buffer (BD Biosciences) for 15 min and then filtered through nylon screen to obtain single cell suspension. Peripheral blood samples were collected by tail bleeding, with RBCs lysed in lysing buffer twice for 15 min. Cells from mouse tissue [BM, spleen and peripheral blood mononuclear cells (MNCs)] were resuspended in DPBS with 0.5% BSA and 0.1% sodium azide for antibody staining. To study hematopoietic stem and progenitor cells, Lineage (Lin) which consists of B220 (RA3-6B2), TER119 (TER119), CD4 (RM4-5), CD8a (53-6.7), Gr-1 (RB6-8C5), and Mac-1 (WT.5), c-Kit (2B8), Sca-1 (D7), Flk2 (A2F10.1) CD34 (RAM34), IL7Rα (SB/199) and FcγRII/III (2.4G2, all antibodies from BD Pharmingen) were used. To study different lineages of blood cells, Gr-1, F4/80 (Invitrogen), B220, CD4, CD8a and CD3 (1F4) were used. CD45.1 (A20) and CD45.2 (104, both from BD Pharmingen) were used for BM transplantation analysis. Integrin α4 and α5 were detected using CD49d (R1-2, eBioscience) and CD49e (5H10-27, BD Pharmingen). 3×10^6^-1×10^7^ BM or spleen cells, or MNCs from 200 µL peripheral blood were incubated with antibodies for 30 min, washed twice with DPBS with 0.5% BSA and 0.1% sodium azide, and then resuspended in DPBS. Dead cells were excluded by 7AAD staining (BD Pharmingen). Cell population analysis was performed on BD FACS LSR II.

### Fluorescence activated cell sorting

Mouse whole BM cells resuspended in DPBS were enriched for c-Kit^+^ cells using CD117 MicroBeads (Miltenyi Biotec, Auburn, CA) according to manufacturer's instructions. c-Kit^+^ cells were stained with anti-mouse c-Kit (Miltenyi Biotec), anti-mouse Sca-1, and Lin antibody cocktail as described above. Primitive hematopoietic stem and progenitor cells were purified using a FACSAria flow cytometer (Becton Dickinson) based on established cell surface phenotypes. LSK cells were sorted for the competitive repopulation and adhesion assays; LK cells were purified for the *in vivo* homing and lodgment assay.

### Cell cycle analysis

1×10^7^ whole BM cells were incubated with 10 µg/ml Hoechst 33342 (Sigma-Aldrich) at 37°C for 45 min, then stained with primitive hematopoietic cell antibodies (Lin, c-Kit and Sca-1) as described above. The stained cells were resuspended in 10% neutral buffered formalin (BDH Chemicals) and incubated at 4°C overnight. To stain for RNA content, Pyronin Y (Polysciences Inc., Warrington, PA) was added to the cells at a final concentration of 0.75 µg/ml and incubated at 4°C for 30 min. Cell cycle status was examined using a BD LSR II flow cytometer.

### Apoptosis assay

1×10^6^ whole BM cells were stained with primitive hematopoietic cell antibodies (Lin, c-Kit and Sca-1) as described above. The stained cells were resuspended in 150 µL 1×Annexin V binding buffer (BD Pharmingen) and incubated with Annexin V and 7AAD (both from BD Pharmingen) for 15 min. Cell apoptosis was examined within an hour using a BD LSR II flow cytometer.

### Colony-forming cell (CFC) assay

Freshly isolated BM cells were mixed thoroughly with 300 µL Iscove's MDM with 2% FBS and 4 mL defined methylcellulose medium M3434 (StemCell Technologies). Cells were then plated in triplicate in six-well plates with 1.1 mL volume at a density of 1×10^4^ BM cells per well. The colonies were then scored at day 10 under an inverted microscope and reported as colony number in specific lineages according to the morphology of colonies described in the manufacturer's instructions.

### Real-time quantitative RT-PCR

To detect *Grp94* knockout efficiency, RNA was extracted from mouse whole BM cells and reverse-transcription was performed as previously described [59]. cDNA samples were analyzed in triplicate with the SYBR Green Supermix (Quanta Biosciences, Gaithersburg, MD) according to manufacturer's instructions. The following primers were used: *Grp94*, 5′-TGG GCC TCT GCT GTG TCC TGC-3′ and 5′-GGC TTT TAC CCA GGT CCT CTT CCA CTG T-3′; *18S RNA*, 5′-ACG GCC GGT ACA GTG AAA C-3′ and 5′-GAG GGA GCT CAC CGG G-3′.

### Competitive repopulation assay

LSK cells were sorted using a FACSAria flow cytometer and 1000 WT or cKO LSK cells (CD45.2) were mixed with 250,000 BM MNCs (CD45.1) which is equivalent to approximately 500 LSK cells, and injected into the tail vein of B6.SJL mice that were lethally irradiated at 9.5Gy approximately 24 hr prior to transplantation. Engraftment levels and multilineage reconstitution were measured in peripheral blood samples obtained from the tail vein of hosts starting week 4.

### *In vivo* homing

Sorted WT and cKO Lin^−^ c-Kit^+^ (LK) cells (∼5×10^5^ to 1×10^6^) were labeled with a green fluorescent dye, carboxyfluorescein diacetate succinimidyl ester (CFSE), and a red fluorescent dye, SNARF-1 (both from Invitrogen), respectively, according to manufacturer's instructions. Labeled cells were injected into the tail vein of non-irradiated *Grp94^F/F^* C57BL6; 129/Sv background littermates. Mice were then sacrificed after 16 hr, and the number of labeled cells was measured in the BM and spleen through the detection of CFSE^+^ and SNARF^+^ cells by flow cytometry.

### *In vivo* lodgment

LK cells were labeled with 5 µM CFSE or 5 µM SNARF cell-labeling solutions and injected into the tail vein of non-irradiated mice, as described above. Tibias and femurs were dissected from recipient mice 16 hr after injection, decalcified for 3 days in Immunocal (Decal Chemical Corporation, Tallman, NY), and embedded in paraffin blocks after processing. A total of 180 bone sections of 5 µm thickness from three recipients were cut and mounted with Vectashield containing 4′,6-diamidino-2-phenylindole (DAPI) (Vector Laboratories, Burlingame, CA). To assess the lodgment of injected cells to the endosteal niche, the numbers of CFSE^+^ (green) or SNARF^+^ (red) cells within two cell diameters from the endosteal surface were counted.

### Adhesion assay

5×10^2^ LSK cells were added to wells coated with fibronectin (1.5 µg/cm^2^, BD Biosciences, Bedford, MA) in cell culture treated 96-well plates and incubated in minimum essential medium (MEM) alpha medium with 10% FBS for 3 hr at 37°C/5% CO_2_ in a humidified atmosphere. Non-adherent cells were washed off with DPBS and adherent cells were visually counted microscopically. To control for nonspecific binding, adhesion to 1% bovine serum albumin (Sigma-Aldrich) was quantified.

### Statistics

Statistical significance was assayed by Student's t test.

## References

[1] Zon LI (2008). Intrinsic and extrinsic control of haematopoietic stem-cell self-renewal.. Nature.

[2] Wagers AJ, Christensen JL, Weissman IL (2002). Cell fate determination from stem cells.. Gene Ther.

[3] Wilson A, Trumpp A (2006). Bone-marrow haematopoietic-stem-cell niches.. Nat Rev Immunol.

[4] Jones DL, Wagers AJ (2008). No place like home: anatomy and function of the stem cell niche.. Nat Rev Mol Cell Biol.

[5] Lymperi S, Ferraro F, Scadden DT (2010). The HSC niche concept has turned 31. Has our knowledge matured?. Ann N Y Acad Sci.

[6] Kiel MJ, Morrison SJ (2008). Uncertainty in the niches that maintain haematopoietic stem cells.. Nat Rev Immunol.

[7] Garrett RW, Emerson SG (2009). Bone and blood vessels: the hard and the soft of hematopoietic stem cell niches.. Cell Stem Cell.

[8] Nilsson SK, Johnston HM, Coverdale JA (2001). Spatial localization of transplanted hemopoietic stem cells: inferences for the localization of stem cell niches.. Blood.

[9] Calvi LM, Adams GB, Weibrecht KW, Weber JM, Olson DP (2003). Osteoblastic cells regulate the haematopoietic stem cell niche.. Nature.

[10] Zhang J, Niu C, Ye L, Huang H, He X (2003). Identification of the haematopoietic stem cell niche and control of the niche size.. Nature.

[11] Yin T, Li L (2006). The stem cell niches in bone.. J Clin Invest.

[12] Cheng T (2008). Toward ‘SMART’ stem cells.. Gene Ther.

[13] Arai F, Hirao A, Ohmura M, Sato H, Matsuoka S (2004). Tie2/angiopoietin-1 signaling regulates hematopoietic stem cell quiescence in the bone marrow niche.. Cell.

[14] Stier S, Ko Y, Forkert R, Lutz C, Neuhaus T (2005). Osteopontin is a hematopoietic stem cell niche component that negatively regulates stem cell pool size.. J Exp Med.

[15] Nilsson SK, Johnston HM, Whitty GA, Williams B, Webb RJ (2005). Osteopontin, a key component of the hematopoietic stem cell niche and regulator of primitive hematopoietic progenitor cells.. Blood.

[16] Adams GB, Chabner KT, Alley IR, Olson DP, Szczepiorkowski ZM (2006). Stem cell engraftment at the endosteal niche is specified by the calcium-sensing receptor.. Nature.

[17] Taichman RS (2005). Blood and bone: two tissues whose fates are intertwined to create the hematopoietic stem-cell niche.. Blood.

[18] Mendez-Ferrer S, Frenette PS (2007). Hematopoietic stem cell trafficking: regulated adhesion and attraction to bone marrow microenvironment.. Ann N Y Acad Sci.

[19] Priestley GV, Scott LM, Ulyanova T, Papayannopoulou T (2006). Lack of alpha4 integrin expression in stem cells restricts competitive function and self-renewal activity.. Blood.

[20] Forsberg EC, Smith-Berdan S (2009). Parsing the niche code: the molecular mechanisms governing hematopoietic stem cell adhesion and differentiation.. Haematologica.

[21] Orford KW, Scadden DT (2008). Deconstructing stem cell self-renewal: genetic insights into cell-cycle regulation.. Nat Rev Genet.

[22] Yuan Y, Shen H, Franklin DS, Scadden DT, Cheng T (2004). In vivo self-renewing divisions of haematopoietic stem cells are increased in the absence of the early G1-phase inhibitor, p18INK4C.. Nat Cell Biol.

[23] Cheng T, Rodrigues N, Shen H, Yang Y, Dombkowski D (2000). Hematopoietic stem cell quiescence maintained by p21cip1/waf1.. Science.

[24] Viatour P, Somervaille TC, Venkatasubrahmanyam S, Kogan S, McLaughlin ME (2008). Hematopoietic stem cell quiescence is maintained by compound contributions of the retinoblastoma gene family.. Cell Stem Cell.

[25] Zhang J, Grindley JC, Yin T, Jayasinghe S, He XC (2006). PTEN maintains haematopoietic stem cells and acts in lineage choice and leukaemia prevention.. Nature.

[26] Yilmaz OH, Valdez R, Theisen BK, Guo W, Ferguson DO (2006). Pten dependence distinguishes haematopoietic stem cells from leukaemia-initiating cells.. Nature.

[27] Hock H, Hamblen MJ, Rooke HM, Schindler JW, Saleque S (2004). Gfi-1 restricts proliferation and preserves functional integrity of haematopoietic stem cells.. Nature.

[28] Zeng H, Yucel R, Kosan C, Klein-Hitpass L, Moroy T (2004). Transcription factor Gfi1 regulates self-renewal and engraftment of hematopoietic stem cells.. EMBO J.

[29] Park IK, Qian D, Kiel M, Becker MW, Pihalja M (2003). Bmi-1 is required for maintenance of adult self-renewing haematopoietic stem cells.. Nature.

[30] Ni M, Lee AS (2007). ER chaperones in mammalian development and human diseases.. FEBS Lett.

[31] Eletto D, Dersh D, Argon Y (2010). GRP94 in ER quality control and stress responses.. Semin Cell Dev Biol.

[32] Maynard JC, Pham T, Zheng T, Jockheck-Clark A, Rankin HB (2010). Gp93, the Drosophila GRP94 ortholog, is required for gut epithelial homeostasis and nutrient assimilation-coupled growth control.. Dev Biol.

[33] Wanderling S, Simen BB, Ostrovsky O, Ahmed NT, Vogen SM (2007). GRP94 is essential for mesoderm induction and muscle development because it regulates insulin-like growth factor secretion.. Mol Biol Cell.

[34] Srivastava PK (2006). Therapeutic cancer vaccines.. Curr Opin Immunol.

[35] Baker-LePain JC, Reed RC, Nicchitta CV (2003). ISO: a critical evaluation of the role of peptides in heat shock/chaperone protein-mediated tumor rejection.. Curr Opin Immunol.

[36] Jockheck-Clark AR, Bowers EV, Totonchy MB, Neubauer J, Pizzo SV (2010). Re-examination of CD91 function in GRP94 (glycoprotein 96) surface binding, uptake, and peptide cross-presentation.. J Immunol.

[37] Randow F, Seed B (2001). Endoplasmic reticulum chaperone gp96 is required for innate immunity but not cell viability.. Nat Cell Biol.

[38] Yang Y, Liu B, Dai J, Srivastava PK, Zammit DJ (2007). Heat shock protein gp96 is a master chaperone for toll-like receptors and is important in the innate function of macrophages.. Immunity.

[39] Liu B, Li Z (2008). Endoplasmic reticulum HSP90b1 (gp96, grp94) optimizes B-cell function via chaperoning integrin and TLR but not immunoglobulin.. Blood.

[40] Staron M, Yang Y, Liu B, Li J, Shen Y (2010). gp96, an endoplasmic reticulum master chaperone for integrins and Toll-like receptors, selectively regulates early T and B lymphopoiesis.. Blood.

[41] Wang M, Ye R, Barron E, Baumeister P, Mao C (2010). Essential role of the unfolded protein response regulator GRP78/BiP in protection from neuronal apoptosis.. Cell Death Differ.

[42] Mao C, Wang M, Luo B, Wey S, Dong D (2010). Targeted mutation of the mouse Grp94 gene disrupts development and perturbs endoplasmic reticulum stress signaling.. PLoS One.

[43] Kuhn R, Schwenk F, Aguet M, Rajewsky K (1995). Inducible gene targeting in mice.. Science.

[44] Lo Celso C, Fleming HE, Wu JW, Zhao CX, Miake-Lye S (2009). Live-animal tracking of individual haematopoietic stem/progenitor cells in their niche.. Nature.

[45] Tzeng YS, Li H, Kang YL, Chen WC, Cheng WC (2011). Loss of Cxcl12/Sdf-1 in adult mice decreases the quiescent state of hematopoietic stem/progenitor cells and alters the pattern of hematopoietic regeneration after myelosuppression.. Blood.

[46] Min IM, Pietramaggiori G, Kim FS, Passegue E, Stevenson KE (2008). The transcription factor EGR1 controls both the proliferation and localization of hematopoietic stem cells.. Cell Stem Cell.

[47] Haylock DN, Williams B, Johnston HM, Liu MC, Rutherford KE (2007). Hemopoietic stem cells with higher hemopoietic potential reside at the bone marrow endosteum.. Stem Cells.

[48] Perkins S, Fleischman RA (1988). Hematopoietic microenvironment. Origin, lineage, and transplantability of the stromal cells in long-term bone marrow cultures from chimeric mice.. Journal of Clinical Investigation.

[49] Jiang Y, Bonig H, Ulyanova T, Chang K, Papayannopoulou T (2009). On the adaptation of endosteal stem cell niche function in response to stress.. Blood.

[50] Wierenga PK, Weersing E, Dontje B, de Haan G, van Os R (2006). Differential role for very late antigen-5 in mobilization and homing of hematopoietic stem cells.. Bone Marrow Transplant.

[51] Adams GB, Scadden DT (2006). The hematopoietic stem cell in its place.. Nat Immunol.

[52] Zhu J, Emerson SG (2002). Hematopoietic cytokines, transcription factors and lineage commitment.. Oncogene.

[53] Zhang K, Wong HN, Song B, Miller CN, Scheuner D (2005). The unfolded protein response sensor IRE1alpha is required at 2 distinct steps in B cell lymphopoiesis.. J Clin Invest.

[54] Brunsing R, Omori SA, Weber F, Bicknell A, Friend L (2008). B- and T-cell development both involve activity of the unfolded protein response pathway.. J Biol Chem.

[55] Czechowicz A, Kraft D, Weissman IL, Bhattacharya D (2007). Efficient transplantation via antibody-based clearance of hematopoietic stem cell niches.. Science.

[56] Fu Y, Lee AS (2006). Glucose regulated proteins in cancer progression, drug resistance and immunotherapy.. Cancer Biol Ther.

[57] Guo W, Lasky JL, Wu H (2006). Cancer stem cells.. Pediatr Res.

[58] Fu Y, Wey S, Wang M, Ye R, Liao CP (2008). Pten null prostate tumorigenesis and AKT activation are blocked by targeted knockout of ER chaperone GRP78/BiP in prostate epithelium.. Proc Natl Acad Sci USA.

[59] Ni M, Zhou H, Wey S, Baumeister P, Lee AS (2009). Regulation of PERK signaling and leukemic cell survival by a novel cytosolic isoform of the UPR regulator GRP78/BiP.. PLoS One.

